# Impact of deep learning based reconstruction algorithms on CT radiomic features of carotid plaques

**DOI:** 10.1002/acm2.70346

**Published:** 2025-11-14

**Authors:** Hanzhe Wang, Jingkai Xu, Chengeng Ye, Aiyun Sun, Jinjin Liu, Shuyang Wang, Xiangwu Zheng, Guoquan Cao

**Affiliations:** ^1^ Department of Radiology The First Affiliated Hospital of Wenzhou Medical University Wenzhou China; ^2^ CT Imaging Research Center GE HealthCare China Shanghai China

**Keywords:** carotid plaque, computed tomography, deep learning, image reconstruction

## Abstract

**Background:**

Radiomics is increasingly applied in carotid plaques analysis to evaluate plaque characteristics and predict cardiovascular risk. However, the influence of different image reconstruction algorithms, particularly deep learning reconstruction (DLIR) and adaptive statistical iterative reconstruction‐Veo (ASIR‐V), on the reproducibility of radiomic features remains poorly understood.

**Purpose:**

To evaluate the impact of DLIR and ASIR‐V on CT radiomic features of carotid plaques.

**Methods:**

76 patients with 104 carotid plaques who underwent head & neck CT angiography were retrospectively enrolled. Images were reconstructed by filtered back projection (FBP), ASIR‐V (30%, 50%, and 80%) and DLIR (DL, DM, and DH). A total of 214 CT‐based radiomic features were organized by statistic family (18 first‐order; 75 texture: 24 GLCM, 14 GLDM, 16 GLRLM, 16 GLSZM, and 5 NGTDM) and transform domain (original and wavelet sub‐bands); 121 features were extracted from wavelet sub‐bands. Features were extracted from both 2D and 3D plaque images. The reliability of feature extraction was evaluated by the intraclass correlation coefficient (ICC).

**Results:**

Different reconstruction algorithms influenced the most radiomic features. The percentages of first‐order, texture, and features in the wavelet domain without statistical difference among 2D and 3D lesions for all seven groups were 0% (0/18), 12.0% (9/75), and 14.9% (18/121), respectively. Compared with FBP, the unaffected features for AV30%, 50%, and 80% decreased from 99.8% and 95.1% to 81.3%, and for DL, DM, and DH from 75.5% and 52.3% to 40.7%. Across statistic families, texture features were the most stable in pairwise comparisons in both the original and wavelet domains. Unaffected features in 2D lesion were larger than 3D lesion. The consistency of first‐order feature in 3D lesion was excellent in both intra‐ and inter‐observer, with ICC values ranging from 0.865 to 1 and 0.790 to 0.999, respectively.

**Conclusion:**

Both ASIR‐V and DLIR algorithms profoundly impact carotid plaque radiomics, with higher strengths exacerbating feature instability. Texture features exhibited superior robustness across all reconstruction protocols. Our findings advocate for a stability‐driven approach to model development: prioritizing robust texture features and employing lower‐strength DLIR are crucial steps to ensure the generalizability of radiomic biomarkers.

## 1 INTRODUCTION

In recent years, with the rapid advancement of imaging technology, radiomics has gradually become an essential tool in medical image analysis. By extracting numerous quantitative features from imaging data, radiomics can capture subtle information that traditional imaging diagnostics may overlook. These features cover aspects like shape, density, and texture,^1^, ^2^ enabling the quantitative assessment of tissue microstructure and revealing the heterogeneity within its microenvironment. Due to these capabilities, radiomics is frequently employed in the diagnosis and prognosis of carotid plaques.^3^, ^4^, ^5^ For example, by analyzing texture features of plaques, radiomics can predict plaque composition, stability, and vulnerability,^6^ as well as forecast cardiovascular events triggered by plaque rupture.

Carotid plaques are significant risk factors for stroke in clinical practice, and their stability and composition are closely associated with the occurrence of cardiovascular and cerebrovascular events.^7^ Therefore, early identification and evaluation of plaque stability are essential for stroke prevention.

In previous studies of carotid plaques, image reconstruction typically employed filtered back projection (FBP) or iterative reconstruction (IR) algorithms. FBP has remained the dominant technique for the past 30 years due to its computational efficiency and accuracy. However, FBP algorithm is highly sensitive to noise, which can severely degrade image quality and diagnostic precision, particularly in low‐dose imaging, thus falling short of meeting the demands for modern low‐dose, high‐quality images. Moreover, FBP struggles to handle more complex imaging scenarios.^8^, ^9^ To address these limitations, IR algorithm, such as the adaptive statistical iterative reconstruction‐Veo (ASIR‐V, GE Healthcare), has been introduced into clinical practice. Although ASIR‐V algorithm can substantially reduce noise, excessive noise suppression caused by higher iteration strength may lead to overly smooth images, leading to an unnatural appearance.^10^, ^11^

With the growing integration of artificial intelligence into the medical field, deep learning‐based image reconstruction algorithms, such as deep learning reconstruction algorithm (DLIR) (TrueFidelity, GE Healthcare), have also been adopted in clinical imaging. Under low radiation dose conditions, DLIR algorithm preserves fine image details, suppresses noise effectively, and enhances both image contrast and clarity.^12^, ^13^ Additionally, it offers three reconstruction strengths (High, Medium, and Low), enabling selection according to specific clinical needs.^14^, ^15^, ^16^, ^17^ As a result, DLIR algorithm is widely used in low‐dose imaging applications, including the evaluation of thoracic^18^ and abdominal lesions,^19^ as well as the assessment of plaques and stenosis in coronary artery^20^ and head and neck vessels.^21^

Most current studies on carotid plaques focus on radiomics, with radiomic features extracted from reconstructed images. However, it remains uncertain whether variations in reconstruction algorithms, in terms of type or strength, may influence CT radiomic features, such as texture characteristics, which could in turn compromise the accuracy of carotid plaque analysis. The aim of this study is to evaluate the impact of different reconstruction algorithms on the consistency and accuracy of CT radiomic features in carotid plaques, with the goal of improving the precision of plaque characterization.

## 2 MATERIALS AND METHOD

### 2.1 Patients

**FIGURE 1:**
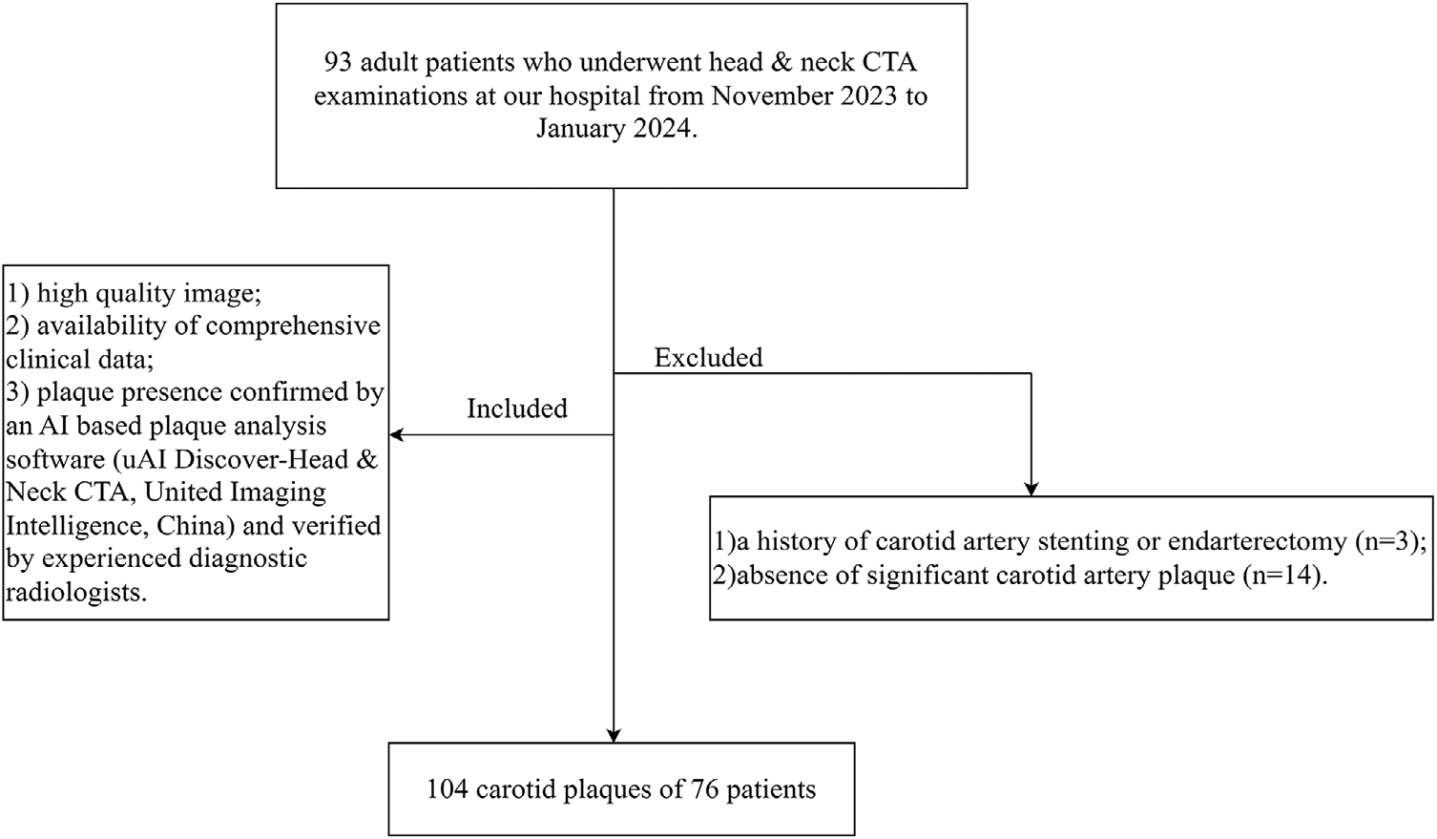
Patient selection flowchart.

Our hospital ethics committee approved this retrospective study and waived patient informed consent. This study included 93 adult patients who underwent head & neck CTA examinations at our hospital from November 2023 to January 2024. Inclusion criteria were as follows: (1) high quality image; (2) availability of comprehensive clinical data; (3) plaque presence confirmed by an AI based plaque analysis software (uAI Discover‐Head & Neck CTA, United Imaging Intelligence, China) and verified by experienced diagnostic radiologists. Exclusion criteria were: (1) a history of carotid artery stenting or endarterectomy; (2) absence of significant carotid plaques (Figure 1).

### 2.2 CT scanning and image reconstruction

All examinations were performed by a 256‐row CT scanner (Revolution Apex CT, GE Healthcare). The carotid CTA were carried out in helical mode with parameters as follows: tube voltage of 120 kVp, pitch of 1.0, slice interval and thickness of 0.625 mm, rotation time of 0.28 s, and scanning ranged from the aortic arch to the cranial vertex. Nonionic‐iodinated contrast agent (350 mgI/mL, Omnipaque 350, GE Healthcare, Shanghai, China) was injected intravenously into the right cubital vein at a rate of 4.0 mL/s using an automatic injector with a bolus of 40 mL and followed by 30‐mL saline flush at the same injection rate. The bolus‐tracking technology was adopted with trigger point at the ascending aorta, trigger threshold of 120 HU and a delay time of 5 s. The raw data were reconstructed into seven sets of images using FBP, adaptive statistical iterative reconstruction‐Veo 30% (AV30%), adaptive statistical iterative reconstruction‐Veo 50% (AV50%), adaptive statistical iterative reconstruction‐Veo 80% (AV80%), DLIR‐Low (DL), DLIR‐Median (DM), and DLIR‐High (DH) algorithms. All images were reconstructed with a slice thickness of 0.625 mm, then transferred to the PACS for anonymization and exported in DICOM format. To ensure consistency, all images and segmentation masks were resampled to isotropic voxels with a size of 1 × 1 × 1 mm^3^ prior to radiomic feature extraction. Image intensities were discretized with a fixed bin width of 25 Hounsfield Units for texture feature computation.

### 2.3 ROI segmentation on CT images and features consistency test

**FIGURE 2:**
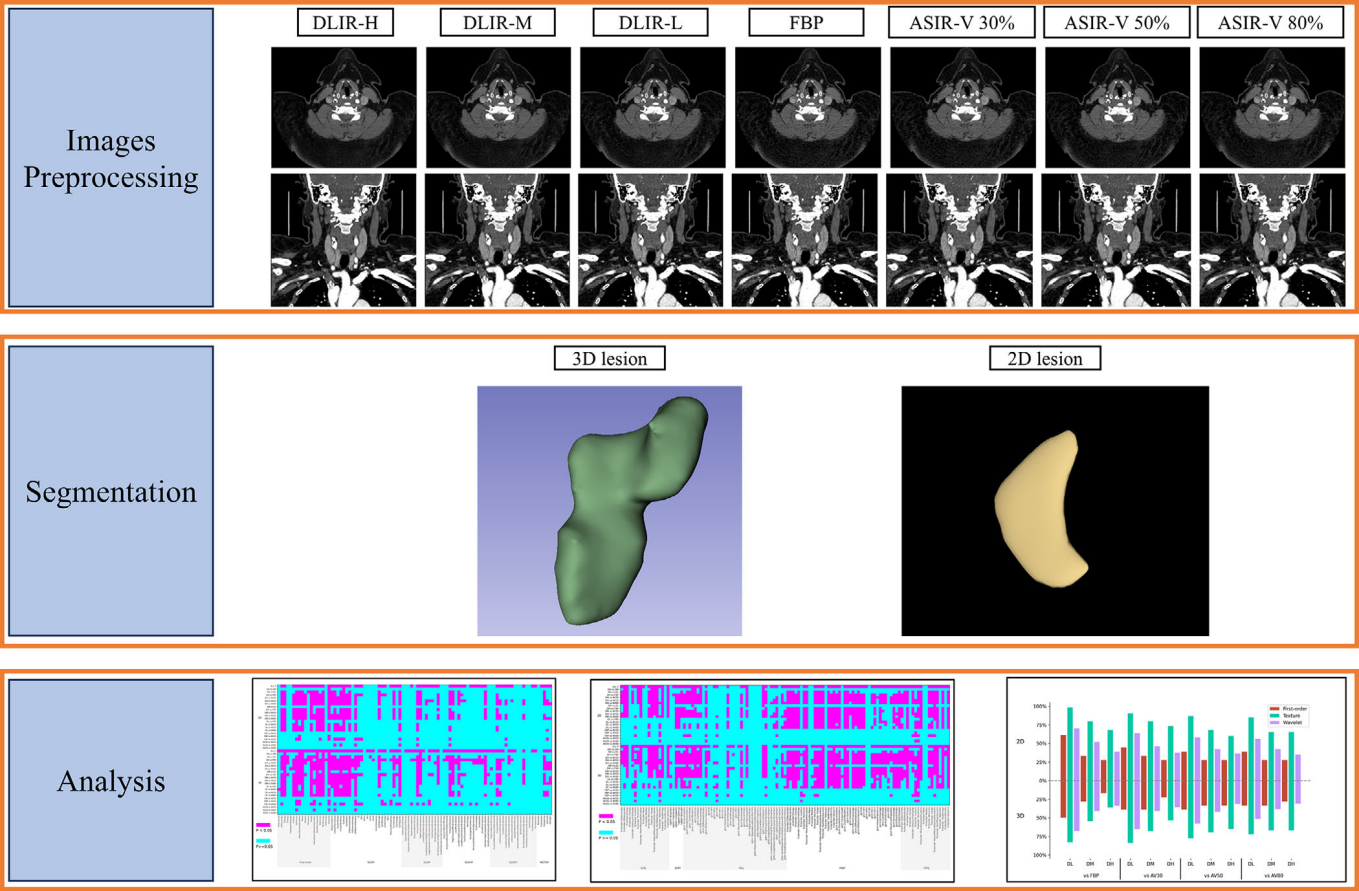
The workflow of study.

Figure 2 illustrates the workflow of this study. DICOM images were imported to the 3D Slicer (version 5.6.1, www.slicer.org). Two experienced radiologists (Radiologist A and B) delineated the region of interest (ROI) on images reconstructed by FBP: (1) for 3D images, the ROI was delineated slice by slice in the axial plane; (2) for 2D images, contouring was performed on the slice displaying the largest plaque cross‐sectional area. This slice‐by‐slice delineation process generated a voxel‐based 3D segmentation volume. To preserve the integrity of the feature data, no surface smoothing, interpolation, or other pre‐processing steps were applied to this label map prior to feature extraction. All radiomic features were computed directly from the original voxel data within this volume. The 3D reconstructions shown for illustrative purposes (e.g., Figure 2) are surface renderings generated by the software. Their visual smoothness is attributable to the software's rendering algorithm and the high *Z*‐axis resolution of the CT scans, and does not indicate any alteration of the underlying data used for analysis. The ROIs were saved as NRRD files and subsequently transferred to the remaining six algorithms to ensure consistent ROI placement. Radiologists (A and B) independently delineated the plaques, and intra‐class correlation coefficients (ICC) were used to assess both intra‐observer and inter‐observer consistency of the extracted features. ICC values > 0.8 were considered to indicate excellent consistency. After 1 month, images from 20 randomly selected patients were reanalyzed to assess both intra‐observer and inter‐observer ICC. Radiologists A and B independently re‐delineated 20 randomly selected images to evaluate intra‐observer and inter‐observer ICC, respectively. To ensure consistency in the delineation of ROIs across different algorithms for the same patient, the ROIs initially outlined on the FBP images were applied to images generated by other six algorithms.

### 2.4 Feature extraction and selection

Radiomic features were extracted using Pyradiomics (version 3.1.0), an open‐source tool integrated into the 3D Slicer software (version 5.6.1, www.slicer.org). A total of 851 features were extracted from each patient, including first‐order features, texture features (gray level co‐occurrence matrix (GLCM) features, gray level dependence matrix (GLDM) features, gray level run length matrix (GLRLM) features, gray level size zone matrix (GLSZM) features, and neighborhood gray tone difference matrix (NGTDM) features), and Wavelet features (Supporting Information). In this study, wavelet denotes the transform domain (features computed on wavelet‐filtered sub‐band images) rather than a homogeneous statistical family. Wavelet‐derived features retain their statistic family labels (first‐order, GLCM, GLDM, GLRLM, GLSZM, and NGTDM) and are interpreted within those families. First‐order features are statistical measures that describe the distribution of pixel or voxel intensity values, including the mean, skewness, and kurtosis.

A pre‐specified, literature‐informed strategy guided feature selection. Six statistical families were included: first‐order, GLCM, GLRLM, GLSZM, GLDM, and NGTDM. Clinically relevant transform‐family strata (e.g., wavelet sub‐bands) were identified through a comprehensive review of carotid plaque radiomics literature, including studies such as Zhang et al.^3^ All features generated under identical transformation and filtering parameters within each family were retained. This rule‐based approach minimized selection bias, reduced analytic degrees of freedom, and resulted in the final set of 214 candidate features, ensuring methodological rigor and reproducibility.

For the 2D analysis, we retained only the axial slice with the largest plaque cross‐sectional area and computed features on that slice using two‐dimensional neighborhoods. Wavelet filtering was performed on this single‐slice input with L/H operations in‐plane (x, y); because the depth equals 1, the through‐plane (z) was handled via boundary padding (e.g., symmetric/reflect). As a result, the outputs retain three‐letter sub‐band labels (e.g., LHL/LHH) by convention to denote filter orientation. Importantly, “2D features” in this study refer strictly to the dimensionality of the feature neighborhood, and these three‐letter tags do not imply the use of a 3D neighborhood during feature computation.

### 2.5 Statistical analysis

Statistical analyses were conducted using Python 3 (version 3.12, http://www.python.org). Continuous variables were expressed as the mean ± standard deviations and nonparametric variables were expressed as the median with interquartile range. The Shapiro–Wilk test was employed to test normality. The repeated measures analysis of variance with least significant difference (LSD) correction was employed to compare the multiple groups of continuous normal variables. The Friedman test with Nemenyi correction was adopted to compare the multiple groups of nonparametric variables. All *p*‐values were obtained from two‐sided tests, with the significance level set at 0.05.

## 3 RESULT

### 3.1 Participants and lesion characteristics

In this study, a total of 76 patients with 104 carotid plaques were included, consisting of 48 males and 28 females, with an age of 64.4 ± 9.9 years (Table 1). The plaques were classified as calcified, non‐calcified, or mixed. A total of 214 radiomic features closely related to carotid plaque characteristics were selected, including 18 first‐order features, 75 texture features (24 GLCM features, 14 GLDM features, 16 GLRLM features, 16 GLSZM features, and 5 NGTDM features), and 121 features derived from wavelet sub‐bands (18 LHL_firstorder, 5 LHH_ngtdm, 24 HLL_glcm, 14 HLL_gldm, 18 HLH_firstorder, 24 HLH_glcm, and 18 HHL_firstorder).

**TABLE 1:** Clinical characteristics of patients.

Characteristic	Value
Number of participants	76
Age (years)	64.4 ± 9.9
Sex (Male/Female)	48/28
Height (cm)	164.1 ± 8.7
Weight (kg)su	65.0 ± 10.0
Body mass index (kg/m^2^)	24.2 ± 3.4
Number of lesions	104

*Note*: Data are means ± standard deviations.

### 3.2 Comparison of first‐order features across reconstruction algorithms

**FIGURE 3:**
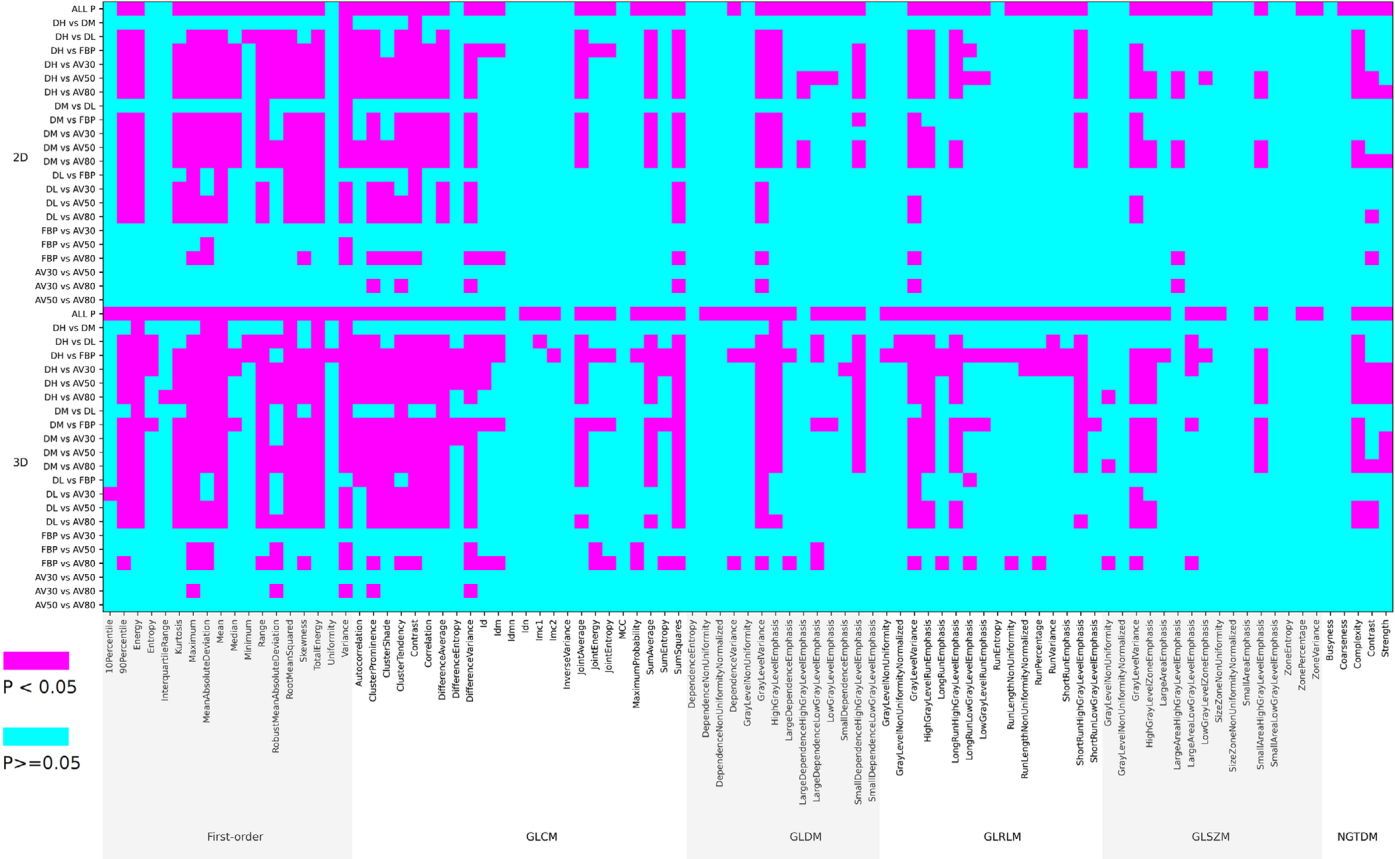
Comparison of *p* values among different reconstruction algorithms, including first‐order and texture features of 2D and 3D lesions. AV30, adaptive statistical iterative reconstruction‐V at 30% strength; AV50, adaptive statistical iterative reconstruction‐V at 50% strength; AV80, adaptive statistical iterative reconstruction‐V at 80% strength; DH, deep learning image reconstruction at high strength; DL, deep learning image reconstruction at low strength; DM, deep learning image reconstruction at medium strength; FBP, filtered back projection.

When comparing feature values across the seven reconstruction algorithms, all first‐order features in 3D lesions showed statistically significant differences. However, in 2D lesions, four features did not demonstrate such significant inter‐algorithm differences (Table 2). In pairwise comparisons between ASIR‐V and FBP algorithms, the proportion of features with no statistically significant difference was 100% (36/36) (AV30% vs. FBP), 83.3% (30/36) (AV50% vs. FBP), and 69.4% (25/36) (AV80% vs. FBP). Similarly, in comparisons between the DLIR and FBP algorithms, this proportion was 22.2%, 30.6%, and 55.6% of features (DH, DM, DL vs. FBP). Approximately 25%, 36.1%, and 41.7% of features (DH, DM, DL vs. AV30%), 30.6%, 30.6%, and 38.9% (DH, DM, DL vs. AV50%), and 27.8%, 30.6%, and 36.1% (DH, DM, DL vs. AV80%) also showed no statistically significant differences (Figure 3).

**TABLE 2:** List of radiomic features demonstrating stability across all seven reconstruction algorithms.

Feature	Dimension	Feature names
First‐order	2D	10Percentile
		Entropy
		InterquartileRange
		Uniformity
Texture	2D/3D	glcm_InverseVariance
		glcm_MCC
		gldm_DependenceEntropy
		gldm_SmallDependenceLowGrayLevelEmphasis
		glszm_SizeZoneNonUniformityNormalized
		glszm_SmallAreaEmphasis
		glszm_SmallAreaLowGrayLevelEmphasis
		glszm_ZoneEntropy, ngtdm_Busyness
Features in the wavelet domain	2D/3D	LHL_firstorder_Entropy
		HLL_glcm_Idm
		HLL_glcm_Imc1
		HLL_glcm_Imc2
		HLL_glcm_InverseVariance
		HLL_glcm_MCC
		HLL_gldm_DependenceEntropy
		HLL_gldm_DependenceNonUniformity
		HLL_gldm_DependenceNonUniformityNormalized
		HLL_gldm_DependenceVariance
		HLL_gldm_GrayLevelNonUniformity
		HLL_gldm_LargeDependenceEmphasis
		HLL_gldm_SmallDependenceEmphasis
		HLH_firstorder_Mean
		HLH_firstorder_Skewness
		HLH_glcm_Idn
		HHL_firstorder_Mean
		HHL_firstorder_Median

### 3.3 Comparison of texture features across reconstruction algorithms

When comparing feature values across the seven algorithm groups, nine features showed no statistically significant differences in either 2D or 3D lesions (Table 2). In subsequent pairwise comparisons, the proportion of texture features with no statistically significant difference between ASIR‐V and FBP algorithms was 100% (150/150) (AV30% vs. FBP), 96.7% (145/150) (AV50% vs. FBP), and 76.7% (115/150) (AV80% vs. FBP) of features showed no statistically significant differences. Similarly, when comparing the DLIR and FBP algorithms, 52%, 67.3%, and 90.7% of features (DH, DM, DL vs. FBP) showed no statistically significant differences. Approximately 63.3%, 64%, and 87.3% of features (DH, DM, DL vs. AV30%), 62.7%, 68.7%, and 82% (DH, DM, DL vs. AV50%), and 66%, 66%, and 78.7% (DH, DM, DL vs. AV80%) also showed no statistically significant differences (Figure 3).

### 3.4 Comparison of features in the wavelet domain across reconstruction algorithms

**FIGURE 4:**
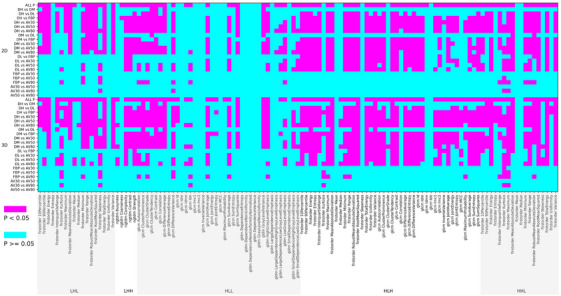
Comparison of *p* values among different reconstruction algorithms for features in the wavelet domain of 2D and 3D lesions. AV50, adaptive statistical iterative reconstruction‐V at 50% strength; AV80, adaptive statistical iterative reconstruction‐V at 80% strength; DH, deep learning image reconstruction at high strength; DL, deep learning image reconstruction at low strength; DM, deep learning image reconstruction at medium strength; FBP, filtered back projection; AV30, adaptive statistical iterative reconstruction‐V at 30% strength.

Out of the 121 features in the wavelet domain, 18 features showed no statistically significant differences across the seven algorithm groups in both 2D and 3D lesions (Table 2), with the wavelet_HLL subgroup accounting for the largest number of features without statistical differences (12 features, *p* > 0.05). Pairwise comparisons between the ASIR‐V and FBP algorithms, the proportion of in the wavelet domain with no statistically significant difference was 99.6% (241/242) (AV30% vs. FBP), 95.6% (232/242) (AV50% vs. FBP), and 85.95% (208/242) (AV80% vs. FBP). Similarly, in comparisons between the DLIR and FBP algorithms, 36.4%, 46.3%, and 69% of features (DH, DM, DL vs. FBP) showed no statistically significant differences. Approximately 36.4%, 43.3%, and 64.5% of features (DH, DM, DL vs. AV30%), 33.9%, 42.1%, and 57.9% (DH, DM, DL vs. AV50%), and 32.6%, 40.1%, and 53.7% (DH, DM, DL vs. AV80%) also showed no statistically significant difference (Figure 4).

### 3.5 Comparison of whole radiomic features

**FIGURE 5:**
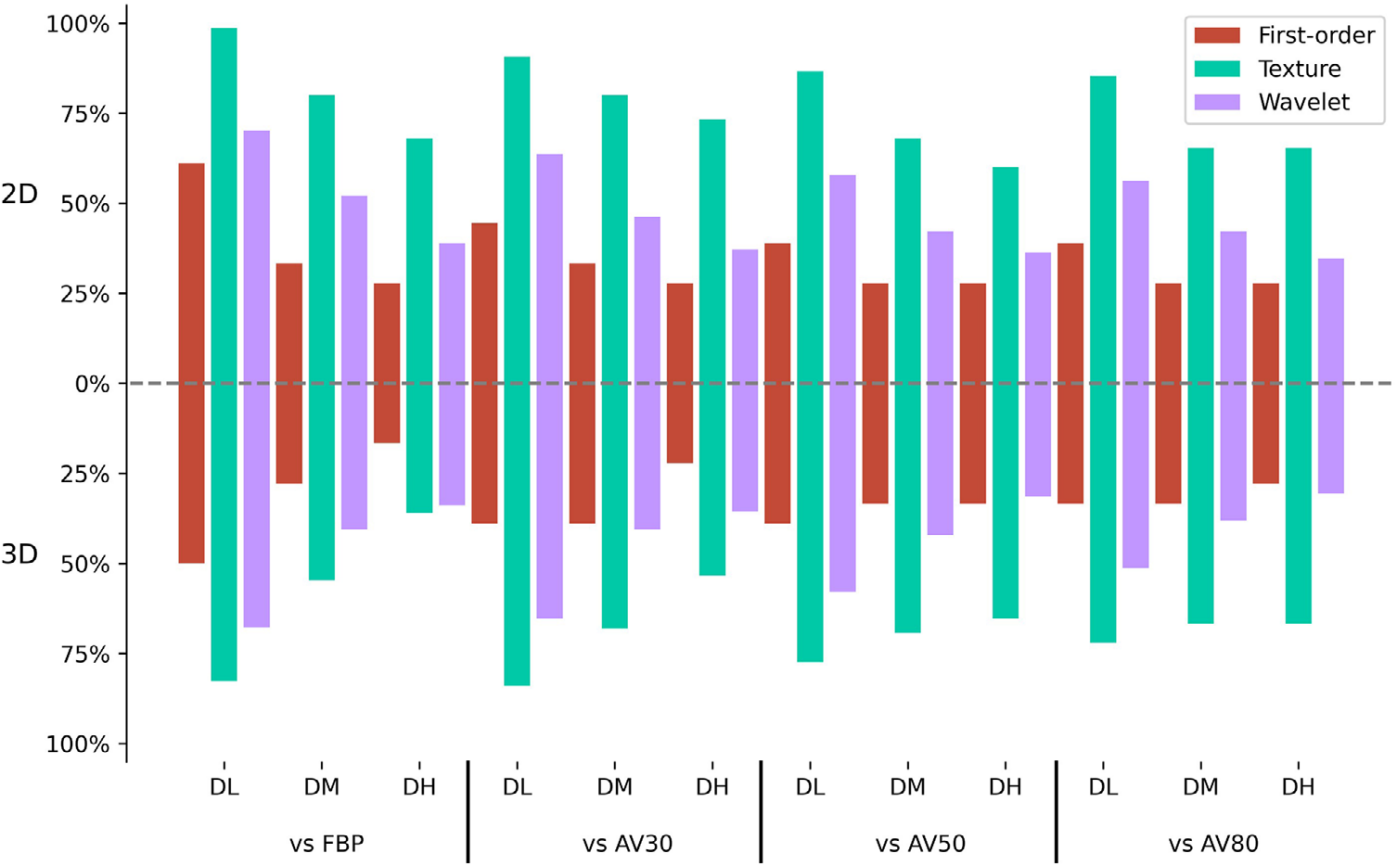
Percentages of radiomic features (including first‐order, texture and features in the wavelet domain) without influence in DLIR vs FBP and ASIR‐V (AV30%, 50%, 80%) of 2D lesions and 3D lesions. AV30, adaptive statistical iterative reconstruction‐V at 30% strength; AV50, adaptive statistical iterative reconstruction‐V at 50% strength; AV80, adaptive statistical iterative reconstruction‐V at 80% strength; DH, deep learning image reconstruction at high strength; DL, deep learning image reconstruction at low strength; DM, deep learning image reconstruction at medium strength; FBP, filtered back projection.

All first‐order features in 2D and 3D lesions across the seven algorithm groups show significant changes, whereas 12% (9/75) of texture features and 14.9% (18/121) of features in the wavelet domain remained unaffected. As the reconstruction strength of the ASIR‐V and DLIR algorithms increased, the proportion of unaffected features decreased progressively. Compared to the FBP algorithm, as reconstruction strength increased from AV30% to AV80%, the proportion of unaffected features declined from 99.8% and 95.1% to 81.3% (Table 3). Similarly, as the DLIR algorithm reconstruction strength increased from low to high, the proportion of unaffected features decreased from 75.5% and 52.3% to 40.7% (Table 3). Additionally, Figure 5 illustrated the extent to which the features of images reconstructed by the DLIR algorithms were affected in comparison to those reconstructed by FBP, AV30%, AV50%, and AV80%.

**TABLE 3:** The proportion of unaffected radiomics features in the comparison of different DLIR (DL, DM, and DH) and different ASIR‐V (AV30, AV50, and AV80) with FBP.

Algorithms (vs FBP)	Number of features test	Features without difference	Proportion (%)
AV30	428	427	99.8%
AV50	428	407	95.1%
AV80	428	348	81.3%
DL	428	323	75.5%
DM	428	224	52.3%
DH	428	174	40.7%

Abbreviations: AV30, adaptive statistical iterative reconstruction‐V at 30% strength; AV50, adaptive statistical iterative reconstruction‐V at 50% strength; AV80, adaptive statistical iterative reconstruction‐V at 80% strength; DH, deep learning image reconstruction at high strength; DL, deep learning image reconstruction at low strength; DM, deep learning image reconstruction at medium strength; FBP, filtered back projection.

### 3.6 Consistency of feature extraction

In feature extraction for 3D lesions, first‐order features demonstrated strong consistency, with intra‐observer ICCs ranging from 0.865 to 1 for repeated delineations by Radiologist A, and inter‐observer ICCs ranging from 0.790 to 0.999 between delineations by Radiologists A and B. The ICCs for texture features and features in the wavelet domain were 0.714–1 and 0.323–1, respectively, both demonstrating lower consistency compared to first‐order features.

## 4 DISCUSSION

This study indicated that texture features were the least influenced by the reconstruction algorithm, followed by features in the wavelet domain and first‐order features, whereas 3D features were more susceptible to algorithmic influence compared to 2D features. Furthermore, as the strength of the DLIR algorithm increased, the disparities between features of the reconstructed images and those generated by FBP and ASIR‐V become increasingly pronounced.

The first‐order features in 2D lesions, including 10Percentile, entropy, interquartileRange, and uniformity, did not show statistical significance across the seven reconstruction algorithms, demonstrating excellent stability. The 10Percentile refers to the 10th percentile of pixel intensity values within an image, while the InterquartileRange is a statistical measure that reflects the difference between the 75th and 25th percentiles. Although different reconstruction algorithms can affect radiomic features, the general statistical features are typically less influenced, as they reflect the overall properties of the image rather than its local details. Entropy quantifies the complexity or uncertainty of the image texture, whereas uniformity evaluates the quantifies of the texture. The calculations of both features are primarily based on the overall pixel distribution. Although variations in local details can impact their results, reconstruction algorithms generally allow these features to maintain the global characteristics and gray‐level stability. Zwanenburg et al. noted that, despite potential impacts on local details from different reconstruction algorithms, standardized statistical features demonstrate good stability and consistency across various devices and reconstruction algorithms.^22^ Therefore, regardless of the reconstruction algorithm employed, researchers can depend on these highly stable features for analysis of plaque diagnosis and follow‐up, providing reliable information on the overall state of the plaques.

Texture features such as GLCM, GLDM, and GLRLM depend on the global statistical distribution of image textures. Although certain detailed features may be impacted by the reconstruction algorithm, they are generally robust when affected by local noise. For example, features like inverse variance, gray level non‐uniformity, and small area low gray level emphasis demonstrated high consistency in 2D features in this study, likely due to the DLIR algorithm's effectiveness in suppressing global texture noise, which reduces these features’ sensitivity to variations in local details. The performance of texture features in 3D lesion is similar to that in 2D lesion. This may be because, in both cases, texture features rely on the overall texture and intensity distribution of the image rather than on individual local details. Kahmann et al. found that the features original_ngtdm_Busyness and original_glcm_MaximumProbability could distinguish between patients with and without at least 50% stenosis, while original_glcm_Idmn, original_gldm_DependenceEntropy, and original_glszm_ZoneEntropy differentiated patients in the 70% stenosis group.^23^ In our study, original_ngtdm_Busyness, original_glcm_Idmn, original_glszm_ZoneEntropy, and original_gldm_DependenceEntropy were unaffected by variations in reconstruction algorithms; original_glcm_MaximumProbability was minimally affected by DLIR, particularly in DM and DL. Although their study focused on coronary plaques and ours on carotid plaques, our findings demonstrate that these features exhibit stability across different reconstruction algorithms, suggesting their potential applicability across plaque types.

Zaccagna et al. noted that skewness was notably higher in vulnerable patients compared to asymptomatic control cases,^24^ suggesting the presence of hyper‐dense objects at any given particular scale.^25^ This observation may reflect microcalcifications, micro‐vessel proliferation, or micro‐ulcerations within the plaque. In our study, we found that compared to the ASIR algorithm, skewness in wavelet features is less affected by the DLIR algorithm and aligns more consistently with the FBP algorithm, particularly in DH and DM.

Texture features demonstrated strong stability in inter‐group comparisons between DLIR and other algorithms, reflecting the strong resistance of texture features to noise and variations in image details. The observed stability of texture features warrants interpretation within the specific engineering of the TrueFidelity algorithm. This deep learning method is documented to preserve a noise texture mimicking that of FBP, a factor that likely explains the high consistency we measured, especially between these two reconstructions. An important implication of our study, therefore, is the provision of a robust evidence base for the use of texture‐based models within this particular technological framework. In contrast, features in the wavelet domain exhibited lower stability, unaffected by reconstruction algorithms. Although not as robust as texture features, features in the wavelet domain still display a certain degree of robustness, and with proper preprocessing and standardization, they also show potential for clinical applications. First‐order features exhibited the lowest stability, and this indicates that first‐order features are highly sensitive to noise and contrast variations during image reconstruction. Additionally, as the strength of DLIR increased, the sensitivity of radiomic features to algorithmic variations also rises with, indicating that while the DLIR effectively reduced image noise, it also influences local details and texture features to varying degrees.^26^ In our study, 2D texture features were more stable than 3D features, likely due to the small structure and high heterogeneity of carotid plaques, which make 3D features more susceptible to subtle changes during algorithmic processing. This finding contrasts with the those of Prezzi and Xue et al.,^27^, ^28^ whose research likely involved larger, more homogeneous lesions, such as those in the liver or rectum, resulting greater stability in 3D features.

We observed that when the type of reconstruction algorithm remained consistent and the strengths were similar, most of the radiomics features (first‐order, texture, and features in the wavelet domain) did not exhibit statistically significant differences. This was evident in comparisons such as DH vs DM, DM vs DL, DL vs FBP, FBP vs AV30%, AV30% vs AV50%, and AV50% vs AV80%, all showing this pattern. We hypothesize that this may be because these algorithms utilizing similar technical principles in the reconstruction process, particularly when comparing different DLIR strengths. The different strengths of the DLIR algorithm are primarily achieved by adjusting the levels of noise suppression and image detail enhancement, while the core reconstruction mechanism remains unchanged. Consequently, although reconstruction strengths differ, the underlying processing logic of the algorithm remains consistent, resulting in a limited impact on radiomic features. IR reconstruction algorithms are typically designed with a high degree of smoothing, gradually improving the handling of noise and image artifacts.^29^ Consequently, the differences between AV30% and AV50%, or AV50% and AV80%, are likely minimal, as the algorithmic changes are incremental and do not cause abrupt shifts in the image.^30^ This smooth transition between algorithms explains the lack of statistically significant differences in radiomic features.

The impact of DLIR on carotid plaque radiomic features became more pronounced as reconstruction strength increased. As DLIR reconstruction progresses from L to H, the number of feature differences in comparisons between the DLIR and FBP algorithms grew. The noise power spectrum (NPS), a metric used to quantify the spatial frequency distribution of image noise, describes noise intensity at various spatial frequencies. Although DLIR exhibits a texture pattern similar to FBP,^21^ increasing reconstruction strength causes a leftward shift in NPS, affecting image texture.^27^, ^31^ This shift occurs because, at higher reconstruction strength, DLIR significantly suppresses noise, enhancing image smoothness and reducing the impact of low‐frequency noise. As noise suppression improves, DLIR refines its handling of subtle image structures and features, resulting in an increasing number of statistically significant feature differences compared to the FBP algorithm.

Our findings highlight a critical challenge in the clinical translation of radiomics: while reconstruction algorithms invariably alter local image details, certain quantitative features—particularly texture metrics—exhibit notable stability and therefore warrant prioritization in model development. This principle has direct implications for applications such as risk stratification of carotid plaques. The pronounced instability of most first‐order features is a case in point. These metrics are often intuitively interpreted as surrogates for fundamental plaque composition. Our analysis, however, reveals their profound dependence on the reconstruction algorithm, especially the strength of DLIR. Consequently, a radiomics model trained on images reconstructed with FBP could yield unreliable and inconsistent predictions when applied to data processed with a high‐strength DLIR protocol. Such algorithmic discordance creates a tangible risk of misclassifying a patient's stroke risk, thereby compromising clinical management. Conversely, the superior robustness of specific texture features offers a pathway toward more dependable clinical tools. As we observed, features including inverse variance, gray level non‐uniformity, and small area low gray level emphasis maintained high consistency in our two‐dimensional analyses. Models built upon such stable metrics, which effectively quantify plaque heterogeneity, are far more likely to retain their predictive performance across different institutions and scanner protocols—a fundamental prerequisite for any biomarker intended for broad clinical adoption. Furthermore, our observation that 2D features were more stable than 3D features suggests that for small, complex structures like carotid plaques, a single‐slice 2D analysis leveraging these robust texture features may currently offer a more reproducible strategy for clinical decision support.

Our study has several limitations. First, as a single‐center study, it may be subject to selection bias. Second, the sample in our study is relatively small, and future validation is needed in larger cohorts. Third, we only investigated the impact of different algorithms on the radiomic features of carotid plaques, without further constructing models to analyze their impacts on model construction and diagnostic accuracy. Fourth, our statistical analysis intentionally forewent correction for multiple comparisons. This approach was chosen to maximize sensitivity for identifying any feature potentially affected by algorithmic variation. In this exploratory context, we contend that the risk of a Type II error (a false negative, i.e., failing to identify an unstable feature) is more detrimental to the development of robust clinical models than a Type I error (a false positive). Consequently, the feature sets identified as non‐robust in our study should be interpreted as a conservative, inclusive list, and the number of truly stable features is likely underestimated. Finally, our study focused on a single‐vendor's equipment. Although this approach allowed for a deep and controlled analysis of the interplay between FBP, ASIR‐V, and DLIR from a single manufacturer, it inherently limits the direct generalizability of our findings to other vendors’ deep learning platforms. Therefore, we strongly advocate for future multi‐vendor, multi‐platform studies to establish a comprehensive “feature reliability map” that can guide the development of truly robust and interoperable radiomic models. In future work, we will continue to expand the sample size and investigate how reconstruction algorithms affect the predictive performance of radiomics features for cerebrovascular events caused by plaques.

## 5 CONCLUSION

Both ASIR‐V and DLIR algorithms profoundly influence carotid plaque radiomics, with the impact magnifying at higher reconstruction strengths. Our findings offer crucial, actionable guidance for developing robust models: texture features consistently demonstrated the highest stability across all reconstruction protocols. Therefore, for building generalizable models in carotid plaque analysis, we strongly recommend prioritizing these stable texture features, along with the specific wavelet‐derived features identified in our study. Specifically, for DLIR, our result suggest that lower reconstruction strengths may be preferable, as they tend to yield a greater number of stable features. Adherence to these principles is paramount to minimizing inter‐algorithm variability, thereby enhancing the generalizability and clinical translation of radiomic biomarkers for carotid plaque characterization.

## AUTHOR CONTRIBUTIONS

**Hanzhe Wang**: Conceptualization; methodology; software; formal analysis; investigation; data curation; writing—original draft preparation; writing review and editing; visualization. **Jingkai Xu**: Software; data curation. **Chengeng Ye**: Formal analysis; data curation. **Aiyun Sun**: Software; methodology; software; formal analysis; writing review and editing. **Jinjin Liu**: writing review and editing. **Shuyang Wang**: Data curation. **Xiangwu Zheng**: Conceptualization; writing review and editing. **Guoquan Cao**: Conceptualization; methodology; formal analysis; writing review and editing, supervision. All authors have read and agreed to the published version of the manuscript.

## CONFLICT OF INTEREST STATEMENT

The authors declare no conflicts of interest.

## ETHICS COMMITTEE APPROVAL

Our hospital ethics committee approved this retrospective study and waived patient informed consent (KY2024‐R313).

## Supporting information



Supporting Information
