# Supplementary material for: Impact of deep learning based reconstruction algorithms on CT radiomic features of carotid plaques
Source: J Appl Clin Med Phys. 2025 Nov 14;26(11):e70346. doi: 10.1002/acm2.70346 (PMC12618174; doi:10.1002/acm2.70346)
Supplement: Supplementary file 1 — Supporting Information [file ACM2-26-e70346-s001.docx]

**Supplemental Materials**

**Supplemental Appendix A: 214 selected features.**

original_firstorder_10Percentile

original_firstorder_90Percentile

original_firstorder_Energy

original_firstorder_Entropy

original_firstorder_InterquartileRange

original_firstorder_Kurtosis

original_firstorder_Maximum

original_firstorder_MeanAbsoluteDeviation

original_firstorder_Mean

original_firstorder_Median

original_firstorder_Minimum

original_firstorder_Range

original_firstorder_RobustMeanAbsoluteDeviation

original_firstorder_RootMeanSquared

original_firstorder_Skewness

original_firstorder_TotalEnergy

original_firstorder_Uniformity

original_firstorder_Variance

original_glcm_Autocorrelation

original_glcm_ClusterProminence

original_glcm_ClusterShade

original_glcm_ClusterTendency

original_glcm_Contrast

original_glcm_Correlation

original_glcm_DifferenceAverage

original_glcm_DifferenceEntropy

original_glcm_DifferenceVariance

original_glcm_Id

original_glcm_Idm

original_glcm_Idmn

original_glcm_Idn

original_glcm_Imc1

original_glcm_Imc2

original_glcm_InverseVariance

original_glcm_JointAverage

original_glcm_JointEnergy

original_glcm_JointEntropy

original_glcm_MCC

original_glcm_MaximumProbability

original_glcm_SumAverage

original_glcm_SumEntropy

original_glcm_SumSquares

original_gldm_DependenceEntropy

original_gldm_DependenceNonUniformity

original_gldm_DependenceNonUniformityNormalized

original_gldm_DependenceVariance

original_gldm_GrayLevelNonUniformity

original_gldm_GrayLevelVariance

original_gldm_HighGrayLevelEmphasis

original_gldm_LargeDependenceEmphasis

original_gldm_LargeDependenceHighGrayLevelEmphasis

original_gldm_LargeDependenceLowGrayLevelEmphasis

original_gldm_LowGrayLevelEmphasis

original_gldm_SmallDependenceEmphasis

original_gldm_SmallDependenceHighGrayLevelEmphasis

original_gldm_SmallDependenceLowGrayLevelEmphasis

original_glrlm_GrayLevelNonUniformity

original_glrlm_GrayLevelNonUniformityNormalized

original_glrlm_GrayLevelVariance

original_glrlm_HighGrayLevelRunEmphasis

original_glrlm_LongRunEmphasis

original_glrlm_LongRunHighGrayLevelEmphasis

original_glrlm_LongRunLowGrayLevelEmphasis

original_glrlm_LowGrayLevelRunEmphasis

original_glrlm_RunEntropy

original_glrlm_RunLengthNonUniformity

original_glrlm_RunLengthNonUniformityNormalized

original_glrlm_RunPercentage

original_glrlm_RunVariance

original_glrlm_ShortRunEmphasis

original_glrlm_ShortRunHighGrayLevelEmphasis

original_glrlm_ShortRunLowGrayLevelEmphasis

original_glszm_GrayLevelNonUniformity

original_glszm_GrayLevelNonUniformityNormalized

original_glszm_GrayLevelVariance

original_glszm_HighGrayLevelZoneEmphasis

original_glszm_LargeAreaEmphasis

original_glszm_LargeAreaHighGrayLevelEmphasis

original_glszm_LargeAreaLowGrayLevelEmphasis

original_glszm_LowGrayLevelZoneEmphasis

original_glszm_SizeZoneNonUniformity

original_glszm_SizeZoneNonUniformityNormalized

original_glszm_SmallAreaEmphasis

original_glszm_SmallAreaHighGrayLevelEmphasis

original_glszm_SmallAreaLowGrayLevelEmphasis

original_glszm_ZoneEntropy

original_glszm_ZonePercentage

original_glszm_ZoneVariance

original_ngtdm_Busyness

original_ngtdm_Coarseness

original_ngtdm_Complexity

original_ngtdm_Contrast

original_ngtdm_Strength

wavelet_LHL_firstorder_10Percentile

wavelet_LHL_firstorder_90Percentile

wavelet_LHL_firstorder_Energy

wavelet_LHL_firstorder_Entropy

wavelet_LHL_firstorder_InterquartileRange

wavelet_LHL_firstorder_Kurtosis

wavelet_LHL_firstorder_Maximum

wavelet_LHL_firstorder_MeanAbsoluteDeviation

wavelet_LHL_firstorder_Mean

wavelet_LHL_firstorder_Median

wavelet_LHL_firstorder_Minimum

wavelet_LHL_firstorder_Range

wavelet_LHL_firstorder_RobustMeanAbsoluteDeviation

wavelet_LHL_firstorder_RootMeanSquared

wavelet_LHL_firstorder_Skewness

wavelet_LHL_firstorder_TotalEnergy

wavelet_LHL_firstorder_Uniformity

wavelet_LHL_firstorder_Variance

wavelet_LHH_ngtdm_Busyness

wavelet_LHH_ngtdm_Coarseness

wavelet_LHH_ngtdm_Complexity

wavelet_LHH_ngtdm_Contrast

wavelet_LHH_ngtdm_Strength

wavelet_HLL_glcm_Autocorrelation

wavelet_HLL_glcm_ClusterProminence

wavelet_HLL_glcm_ClusterShade

wavelet_HLL_glcm_ClusterTendency

wavelet_HLL_glcm_Contrast

wavelet_HLL_glcm_Correlation

wavelet_HLL_glcm_DifferenceAverage

wavelet_HLL_glcm_DifferenceEntropy

wavelet_HLL_glcm_DifferenceVariance

wavelet_HLL_glcm_Id

wavelet_HLL_glcm_Idm

wavelet_HLL_glcm_Idmn

wavelet_HLL_glcm_Idn

wavelet_HLL_glcm_Imc1

wavelet_HLL_glcm_Imc2

wavelet_HLL_glcm_InverseVariance

wavelet_HLL_glcm_JointAverage

wavelet_HLL_glcm_JointEnergy

wavelet_HLL_glcm_JointEntropy

wavelet_HLL_glcm_MCC

wavelet_HLL_glcm_MaximumProbability

wavelet_HLL_glcm_SumAverage

wavelet_HLL_glcm_SumEntropy

wavelet_HLL_glcm_SumSquares

wavelet_HLL_gldm_DependenceEntropy

wavelet_HLL_gldm_DependenceNonUniformity

wavelet_HLL_gldm_DependenceNonUniformityNormalized

wavelet_HLL_gldm_DependenceVariance

wavelet_HLL_gldm_GrayLevelNonUniformity

wavelet_HLL_gldm_GrayLevelVariance

wavelet_HLL_gldm_HighGrayLevelEmphasis

wavelet_HLL_gldm_LargeDependenceEmphasis

wavelet_HLL_gldm_LargeDependenceHighGrayLevelEmphasis

wavelet_HLL_gldm_LargeDependenceLowGrayLevelEmphasis

wavelet_HLL_gldm_LowGrayLevelEmphasis

wavelet_HLL_gldm_SmallDependenceEmphasis

wavelet_HLL_gldm_SmallDependenceHighGrayLevelEmphasis

wavelet_HLL_gldm_SmallDependenceLowGrayLevelEmphasis

wavelet_HLH_firstorder_10Percentile

wavelet_HLH_firstorder_90Percentile

wavelet_HLH_firstorder_Energy

wavelet_HLH_firstorder_Entropy

wavelet_HLH_firstorder_InterquartileRange

wavelet_HLH_firstorder_Kurtosis

wavelet_HLH_firstorder_Maximum

wavelet_HLH_firstorder_MeanAbsoluteDeviation

wavelet_HLH_firstorder_Mean

wavelet_HLH_firstorder_Median

wavelet_HLH_firstorder_Minimum

wavelet_HLH_firstorder_Range

wavelet_HLH_firstorder_RobustMeanAbsoluteDeviation

wavelet_HLH_firstorder_RootMeanSquared

wavelet_HLH_firstorder_Skewness

wavelet_HLH_firstorder_TotalEnergy

wavelet_HLH_firstorder_Uniformity

wavelet_HLH_firstorder_Variance

wavelet_HLH_glcm_Autocorrelation

wavelet_HLH_glcm_ClusterProminence

wavelet_HLH_glcm_ClusterShade

wavelet_HLH_glcm_ClusterTendency

wavelet_HLH_glcm_Contrast

wavelet_HLH_glcm_Correlation

wavelet_HLH_glcm_DifferenceAverage

wavelet_HLH_glcm_DifferenceEntropy

wavelet_HLH_glcm_DifferenceVariance

wavelet_HLH_glcm_Id

wavelet_HLH_glcm_Idm

wavelet_HLH_glcm_Idmn

wavelet_HLH_glcm_Idn

wavelet_HLH_glcm_Imc1

wavelet_HLH_glcm_Imc2

wavelet_HLH_glcm_InverseVariance

wavelet_HLH_glcm_JointAverage

wavelet_HLH_glcm_JointEnergy

wavelet_HLH_glcm_JointEntropy

wavelet_HLH_glcm_MCC

wavelet_HLH_glcm_MaximumProbability

wavelet_HLH_glcm_SumAverage

wavelet_HLH_glcm_SumEntropy

wavelet_HLH_glcm_SumSquares

wavelet_HHL_firstorder_10Percentile

wavelet_HHL_firstorder_90Percentile

wavelet_HHL_firstorder_Energy

wavelet_HHL_firstorder_Entropy

wavelet_HHL_firstorder_InterquartileRange

wavelet_HHL_firstorder_Kurtosis

wavelet_HHL_firstorder_Maximum

wavelet_HHL_firstorder_MeanAbsoluteDeviation

wavelet_HHL_firstorder_Mean

wavelet_HHL_firstorder_Median

wavelet_HHL_firstorder_Minimum

wavelet_HHL_firstorder_Range

wavelet_HHL_firstorder_RobustMeanAbsoluteDeviation

wavelet_HHL_firstorder_RootMeanSquared

wavelet_HHL_firstorder_Skewness

wavelet_HHL_firstorder_TotalEnergy

wavelet_HHL_firstorder_Uniformity

wavelet_HHL_firstorder_Variance
